# me101 is a new allele of rad-51

**DOI:** 10.17912/micropub.biology.000107

**Published:** 2019-04-26

**Authors:** Baptiste Roelens, Karl A Zawadzki, Anne M Villeneuve

**Affiliations:** 1 Departments of Developmental Biology and Genetics, Stanford University School of Medicine

**Figure 1.**
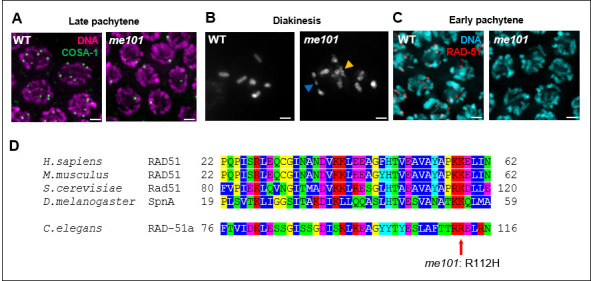
**A.** Detection of crossover site marker GFP::COSA-1 (green) and DNA counterstaining (magenta) in wild-type (left) and me101 mutant (right) late pachytene nuclei. **B.** DAPI-stained chromosomes in oocytes of the indicated genotype at diakinesis, the last stage of meiotic prophase; while six pairs of attached homologs are consistently detected at this stage in wild-type oocytes, poorly condensed chromosomes, chromosome fragments (blue arrowhead) and/or chromosome aggregates (yellow arrowhead) are observed in me101 mutant meiocytes. **C.** The recombinase RAD-51 (red) is detected in wild-type but absent in me101 mutant early pachytene nuclei. Scale bar in panels A-C represents 2µm. **D.** ClustalW alignment of protein sequences of RAD-51 orthologs from the indicated species. The me101 mutation induces a substitution in a conserved acidic residue.

## Description

The *me101* allele was isolated in a genetic screen for mutants with an altered number of GFP::COSA-1 foci, which mark the sites of crossovers in *C. elegans* germ cells (Rosu *et al.* 2013). After multiple rounds of outcrossing, we confirmed that *me101* mutants were defective in some aspects of meiotic prophase, as late pachytene *me101* mutant meiocytes failed to form the six GFP::COSA-1 foci observed in wild-type late pachytene meiocytes (Fig 1.A). We also observed structural defects ranging from chromosome fragmentation to the formation of chromosome aggregates in *me101* diakinesis-stage oocytes (Fig. 1B), suggesting a defect in some aspect of the DNA damage response. Further, 100% of eggs laid by *me101* mutant hermaphrodites are inviable. We then assessed the localization of the recombinase RAD-51, an essential component of the homologous recombination machinery that is required for the repair of DNA breaks and the maintenance of genome integrity during meiosis (Rinaldo *et al.* 2002; Alpi *et al.* 2003); no RAD-51 foci were observed in the gonads of *me101* mutants (Fig 1C). Sequencing of the *rad-51* locus in the *me101* mutant revealed a single G to A substitution (IV:10283785 from WS269), leading to an Arginine to Histidine substitution in a conserved residue (Fig. 1D). Failure to detect RAD-51 foci in the *me101* mutant indicates that this residue is important for the loading and/or the stability of the RAD-51 protein.

## Methods

Cytology: Immunofluorescent detection of GFP::COSA-1 and RAD-51 was performed as described in (Martinez-Perez and Villeneuve 2005) using a mouse anti-GFP antibody (Sigma-Aldrich #11814460001) and a rabbit anti-RAD-51 antibody (Colaiacovo *et al.* 2003).

## Reagents

Strains:

AV727 *meIs8[pie-1p::gfp::cosa-1 + unc-119(+)] II ; ltIs37[pie-1p::mCherry::his-58 + unc-119(+)] IV ; ltIs38[pie-1p::gfp::ph(PLC1delta1) + unc-119(+)]*

AV880 *meIs8[pie-1p::gfp::cosa-1 + unc-119(+)] II ; rad-51(me101) ltIs37[pie-1p::mCherry::his-58 + unc-119(+)] / nT1[qIs51] IV ; +/nT1 V; ltIs38[pie-1p::gfp::ph(PLC1delta1) + unc-119(+)]*

## References

[R1] Alpi A, Pasierbek P, Gartner A, Loidl J (2003). Genetic and cytological characterization of the recombination protein RAD-51 in Caenorhabditis elegans.. Chromosoma.

[R2] Colaiácovo MP, MacQueen AJ, Martinez-Perez E, McDonald K, Adamo A, La Volpe A, Villeneuve AM (2003). Synaptonemal complex assembly in C. elegans is dispensable for loading strand-exchange proteins but critical for proper completion of recombination.. Dev Cell.

[R3] Martinez-Perez E, Villeneuve AM (2005). HTP-1-dependent constraints coordinate homolog pairing and synapsis and promote chiasma formation during C. elegans meiosis.. Genes Dev.

[R4] Rinaldo C, Bazzicalupo P, Ederle S, Hilliard M, La Volpe A (2002). Roles for Caenorhabditis elegans rad-51 in meiosis and in resistance to ionizing radiation during development.. Genetics.

[R5] Rosu S, Zawadzki KA, Stamper EL, Libuda DE, Reese AL, Dernburg AF, Villeneuve AM (2013). The C. elegans DSB-2 protein reveals a regulatory network that controls competence for meiotic DSB formation and promotes crossover assurance.. PLoS Genet.

